# Robot-assisted surgery for rectal cancer in situs inversus totalis: a case report

**DOI:** 10.1093/jscr/rjag144

**Published:** 2026-03-12

**Authors:** Munenori Takaoka, Atsushi Urakami, Kazuki Matsushita, Akihisa Akagi, Takashi Urano, Masaki Matsubara, Kaori Shigemitsu, Tomoki Yamatsuji

**Affiliations:** Department of General Surgery, Kawasaki Medical School General Medical Center, 2-6-1 Nakasange, Kita-ku, Okayama 700-8505, Japan; Department of General Surgery, Kawasaki Medical School General Medical Center, 2-6-1 Nakasange, Kita-ku, Okayama 700-8505, Japan; Department of General Surgery, Kawasaki Medical School General Medical Center, 2-6-1 Nakasange, Kita-ku, Okayama 700-8505, Japan; Department of General Surgery, Kawasaki Medical School General Medical Center, 2-6-1 Nakasange, Kita-ku, Okayama 700-8505, Japan; Department of General Surgery, Kawasaki Medical School General Medical Center, 2-6-1 Nakasange, Kita-ku, Okayama 700-8505, Japan; Department of General Surgery, Kawasaki Medical School General Medical Center, 2-6-1 Nakasange, Kita-ku, Okayama 700-8505, Japan; Department of General Surgery, Kawasaki Medical School General Medical Center, 2-6-1 Nakasange, Kita-ku, Okayama 700-8505, Japan; Department of General Surgery, Kawasaki Medical School General Medical Center, 2-6-1 Nakasange, Kita-ku, Okayama 700-8505, Japan

**Keywords:** situs inversus totalis (SIT), rectal cancer, robot-assisted surgery, robotic surgery

## Abstract

Situs inversus totalis (SIT) is a relatively rare congenital malformation; and particular emphasis should be placed on its anatomical recognition during surgery. Here, we report a rare case of rectal cancer in SIT, treated with robot-assisted surgery (RAS). A 71-year-old man with SIT was referred to our department with advanced rectal cancer. Robot-assisted anterior resection, using the DaVinci Xi surgical system, was performed. The ports were arranged in a normal mirror image with the camera port (2nd arm) inside the lower left abdomen. The robotic procedure of total mesenteric excision with D3 lymph node dissection was achieved, and re-roll-in of the robotic arms was implemented for anastomosis, using the double-stapling technique and additional suture reinforcement at the anastomotic site. RAS was conducted with safety and efficiency; his postoperative course was uneventful and no adverse event was observed.

## Introduction

Situs inversus totalis (SIT) is a congenital anomaly, occurring in approximately one in 10 000–20 000 adults, where the organs in the chest and abdomen are completely reversed [1]. While SIT itself generally lacks pathological significance, it necessitates careful anatomical consideration during surgical procedures. Notably, there is a paucity of literature regarding robotic-assisted surgery (RAS) for rectal cancer in SIT; and further research is required to ascertain whether RAS offers advantages over traditional surgical methods in this context. In this report, we present a case involving a patient with rectal cancer in SIT who was treated by RAS.

## Case report

**Figure 1:**
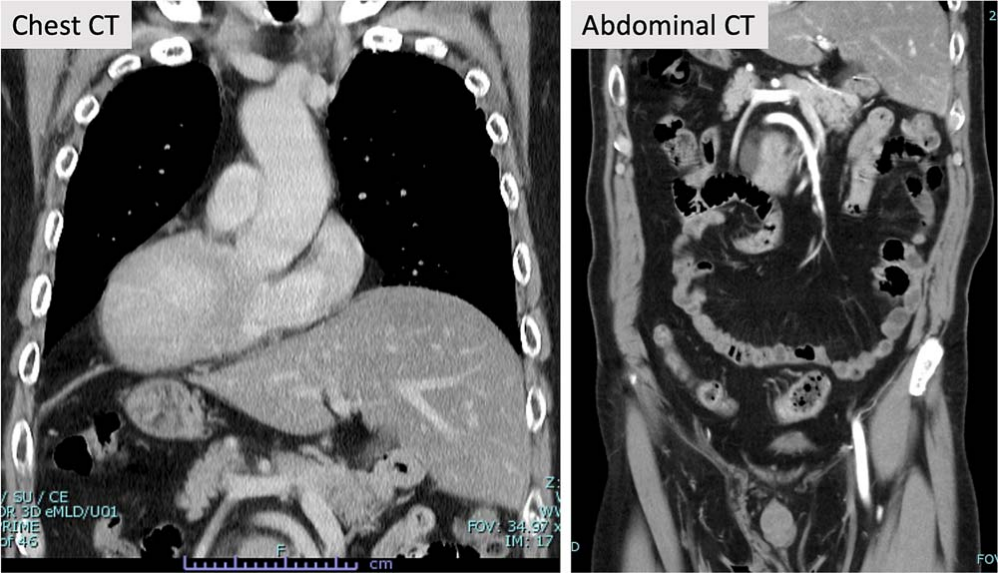
Contrast-enhanced CT. Contrast-enhanced CT revealed SIT. Other abnormalities in the blood vessels were not evident in the chest and abdomen.

**Figure 2:**
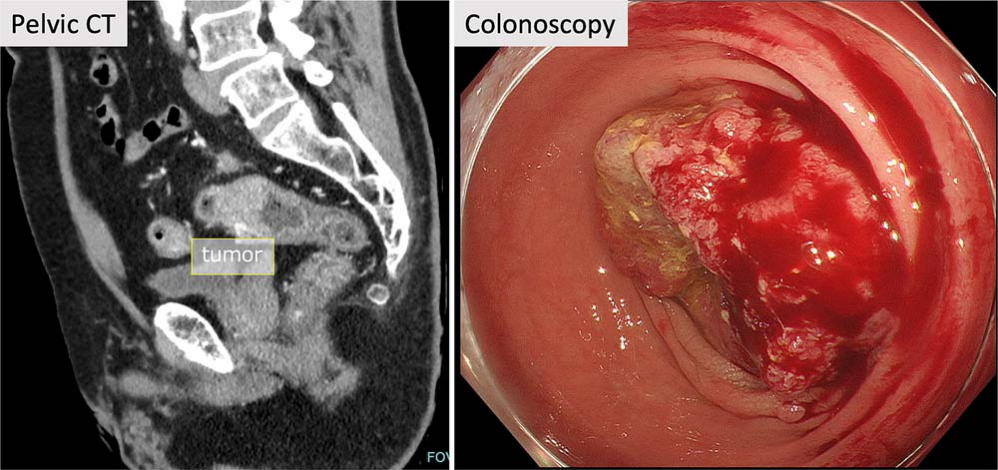
Pelvic CT and colonoscopy. Pelvic CT showed a tumor in the upper rectum, and rectal stenosis with the tumor was observed in colonoscopy.

A 71-year-old male patient presented to our hospital with complaints of abnormal bowel movements. He had a history of laparoscopic cholecystectomy and had been previously diagnosed with congenital SIT (Fig. 1). Pelvic computed tomography (CT) showed a tumor in the upper rectum (Fig. 2). Colonoscopy revealed rectal stenosis due to the tumor (Fig. 2); and biopsy confirmed a diagnosis of moderately differentiated adenocarcinoma. Preoperative assessments indicated an absence of distant metastasis; therefore, robot-assisted surgery (RAS) was planned, despite the presence of SIT.

**Figure 3:**
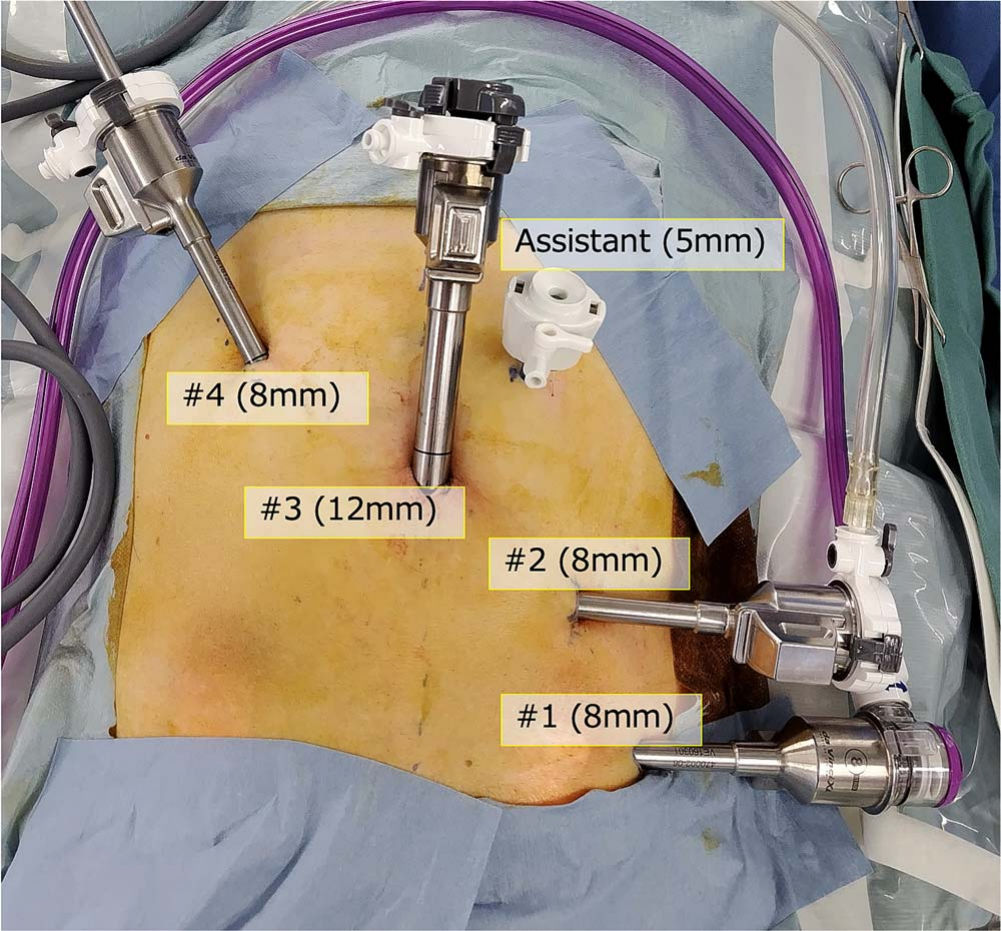
Port position. Five ports including the assistant trocar were arranged in the mirror setting of conventional RAS.

The RAS of the rectal cancer, using the DaVinci Xi surgical system, was performed. Five ports, including the trocar as assistant, were arranged in the usual mirror setting. Port#1 (8 mm) was located outside of the left abdomen. Port #2 (8 mm), located in the inner left lower abdomen, was designated as a camera port, resulting in port #3 to the umbilics (12 mm) and port #4 (8 mm) in the inner right upper abdomen, used for right-hand equipment such as monopolar curved scissors, a linear stapler, needle driver and tip-up fenestrated grasper (Fig. 3).

The assistant port (5 mm) was placed in the upper left abdomen; and a 30° camera was used. The settings for the other devices surrounding the patient were the same as usual. His cart was docked on the right side; and adhesions were not observed in the operative field.

**Figure 4:**
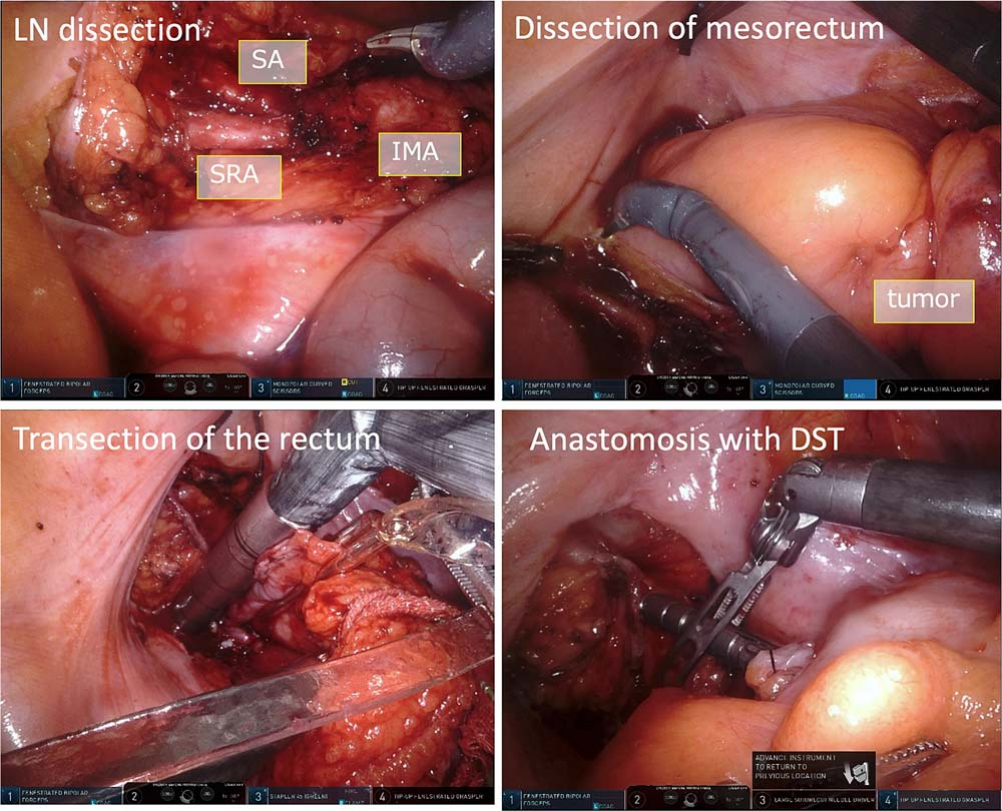
Intraoperative images. The IMA, SRA, and SA were exposed and lymph node dissection was done. The mesorectum was dissected and mobilized. To transect the rectum, we used the robotic linear stapler, and anastomosis was performed using DST.

The console surgeon operated with the fenestrated bipolar forceps (#1 arm) in his left hand, and monopolar curved scissors (#3 arm) in his right hand. A tip-up fenestrated grasper (#4 arm) was used to ensure the surgical field in his right hand. The robotic procedure was initiated with the cutting of the peritoneum behind the inferior mesenteric artery (IMA) root. The IMA, superior rectal artery (SRA), and sigmoidal artery (SA) were exposed, and lymph node dissection (D3) was performed (Fig. 4).

The SRA and SA were clipped and divided. The sigmoidal mesocolon and mesorectum were dissected and mobilized from the medial side to the lateral side (Fig. 4). To transect the rectum, a robotic linear stapler (Sureform 45 mm green) was inserted through the 12 mm trocar (#3 port) in the umbilicus (Figs 3 and 4). Once the patient cart was rolled-out and the rectum was resected, re-roll-in was performed for anastomosis. This was performed using a double stapling technique (DST), using a circular stapler (Fig. 4). The total operation time was 304 min; the console time was 220 min; and the bleeding volume was 5 mL. The patient was discharged without any adverse event. Histological examination revealed that the cancer stage was pT3N1M0 pStageIIIb (UICC, 8th edition) with negative margins.

## Discussion

SIT is a congenital malformation in which the anatomical positions of the chest and abdominal organs are reversed. It has little pathological significance, particular emphasis should be placed on the position of ports and anatomical recognition during surgery. The important points to remember for safety are as follows: 1) Ensure sufficient preoperative evaluation of cardiovascular malformations and abnormalities in blood vessels; 2) Reverse the usual setting positions of the instruments and the monitor; and 3) Perform surgical maneuvers with careful attention to the mirror image, so as not to misidentify the anatomy [2]. However, because of pelvic symmetry, dissection and mobilization of the mesorectum and rectal transection on the anorectal side of the tumor may be performed as in a normal rectal cancer operation.

In the case of laparoscopic surgery, the use of left-hand instruments characterizes an exact mirror image of the technique used in normal patients. Otherwise, the robotic system stabilizes procedures, filters tremors, and provides a 3D view of the operative field. Furthermore, multi-joint instruments allow surgeons to operate flexibly and precisely with both hands. These advantageous features of RAS enable dissection, which is a complicated and precise procedure; and ensure surgically safe outcomes for any anatomical anomaly [3]. It has been reported that changing surgeons’ standing positions during surgery in SIT is effective in laparoscopic surgery [4, 5]. We selected the patient’s opposite cart position and port positions. The procedures were performed from the left side and did not differ from that of usual operations. During the entire surgery, we did not feel much discomfort from the SIT, and we felt minimal stress using these settings, which may be because the procedure was performed by magnifying the local area of the surgical field through robotic surgery.

Kato *et al.* [6] summarized five cases of RAS for colorectal cancer in SIT [3, 7–9]. Each report had different port positions and procedures, and acknowledged the efficacy and safety of RAS; and the benefits of MIS. Here, we used the DaVinci Xi surgical system and selected different port positions. Although we felt some maladjustment at the beginning of the surgery, we became easily used to the advanced technologies provided by the robotic system, and the operation was performed safely.

In conclusion, because of the advanced technologies of the DaVinci system, RAS for rectal cancer in SIT may be a safer and more effective approach than previous surgical techniques.
